# The Effects of Immunosuppression on the Lung Microbiome and Metabolites in Rats

**DOI:** 10.3389/fmicb.2022.817159

**Published:** 2022-02-14

**Authors:** Huiwei Dong, Rong Tan, Zhengshan Chen, Lifang Wang, Yuanyuan Song, Min Jin, Jing Yin, Haibei Li, Junwen Li, Dong Yang

**Affiliations:** Tianjin Institute of Environmental and Operational Medicine, Tianjin, China

**Keywords:** immunosuppression, pneumonia, microbiome, metabolites, metabolic pathway

## Abstract

Immunosuppressed patients are more likely to suffer from pneumonia, especially *Streptococcus* and *Enterobacter* pneumonia. Studies have demonstrated the existence of a complex and dynamic microbiota on the surface of human respiratory epithelial cells, both in healthy and diseased states. However, it is not clear whether the pneumonia in immunosuppressed patients is caused by inhaled oropharyngeal pathogens or abnormal proliferation of pulmonary proteobacteria. In this study, immunosuppressed model was made by intraperitoneal injection of cyclophosphamide and oropharyngeal saliva aspiration was simulated by oral and pharyngeal tracheal instillation of sterilized phosphate buffered saline (PBS). Furthermore, the effects of immunosuppression on the lung microbial community and its metabolism were investigated using 16S rRNA gene sequencing and liquid chromatography-mass spectrometry (LC-MS) metabolomics analysis. The 16S rRNA gene sequencing results showed that immunosuppression alone did not change the composition of pulmonary bacteria. Moreover, although the bacteria brought by sterilized PBS from oropharynx to lower respiratory tract changed the composition of the microflora in healthy and immunosuppressed rats, the change in the latter was more obvious. Metabolomic analysis revealed that the levels of pulmonary metabolites were disturbed in the immunosuppressed rats. The altered lung microbiota, including *Streptococcaceae* and *Enterobacteriaceae*, showed significant positive correlations with pulmonary metabolites. Our study suggested that the source of the pathogens of pneumonia in immunosuppressed rats was *via* inhalation and explored the relationship between lung microbiome and metabolites in immunosuppressed rats. Our results provide the basis for the development of prevention and treatment strategies for pneumonia.

## Highlights

- A rat immunosuppression model was made by cyclophosphamide injection.
- 16S rRNA gene sequencing and metabolomics analysis were performed.
- Pulmonary metabolite levels were disturbed in the immunosuppressed rats.
- The altered lung microbiota correlated significantly with pulmonary metabolites.
- Pneumonia pathogens in immunosuppressed rats entered *via* inhalation.

## Introduction

The aging of the population has resulted in an increase in the prevalence of chronic diseases and the long-term use of immunosuppressive drugs for treatment (Furman et al., 2004; Cillóniz et al., 2013). Thus, the number of immunosuppressed patients suffering from pneumonia is significantly increasing (Furman et al., 2004; Cillóniz et al., 2013). According to Di Pasquale’s research, about 20% of 3,700 hospitalized patients with pneumonia were immunosuppressed (Di Pasquale et al., 2019). According to Jain’s research, about 30% of ICU admissions of patients with cancer, most of whom are immunosuppressed, had bacterial pneumonia (Jain et al., 2015). Immunosuppression increases the risk and severity of bacterial infections (Morrison, 2014), especially severe respiratory infections, which might lead to septicemia and hypoxemic acute respiratory failure or even death (Azoulay et al., 2019). Therefore, it is necessary to better understand the pathogenic process of immunosuppression-related pneumonia.

With the technological progress of high-throughput molecular sequencing, studies have demonstrated the existence of a dynamic and complex microbial community on the surface of human respiratory epithelial cells, both in healthy and disease states (Dickson and Huffnagle, 2015). Although the respiratory tract microbes are not as abundant as those in the intestinal tract, the rich vascular structure of the lung will directly expose the microbial components of the lower respiratory tract to the bloodstream, thus causing a more direct response of the immune system (O’Dwyer et al., 2016; Suresh and Shimoda, 2016; Cho and Stout-Delgado, 2020). Compared with healthy people, studies have shown that the structure of the pulmonary microflora in patients who suffer from chronic obstructive pulmonary disease changed, the diversity increased, the abundance of *Proteus* and *Actinomycetes* was higher, and the abundance of *Firmicutes* and *Bacteroides* was lower than that of healthy people (Huang et al., 2010, 2014; Sze et al., 2014). Furthermore, the sputum samples of the patients contained more *Haemophilus influenzae*. The changes of these microflora might cause changes in the immune state of the lungs, thereby damaging bronchioles and alveolar tissues, reducing the synthesis of alveolar surfactants, and thus promoting the occurrence of emphysema (Reale et al., 2012). In patients with asthma, *Proteobacteria*, especially conditional pathogens, such as *Haemophilus*, *Moraxella*, and *Neisseria*, have been shown to be significantly increased, while *Bacteroides*, especially *Prevotella*, were significantly decreased compared with those in healthy people (Durack et al., 2018; Sokolowska et al., 2018). The colonization mode of respiratory tract flora is closely related to the acute attack, severity, and therapeutic effect of asthma (Chung, 2017). Thus, respiratory microorganisms might directly affect the health of the host.

The growth of respiratory tract microbes is affected by the environmental conditions of the respiratory tract, such as temperature, pH, oxygen tension, nutrient availability, and activation of host inflammatory cells. In addition, there are bacteria in the environment in which we live, especially in the polluted air, which is a repository of all kinds of microorganisms, which can affect pulmonary bacteria in various ways (Stephens, 2016; Chng et al., 2020). Exposure of the lower respiratory tract to microorganisms usually occurs in individuals, such as inhalation of microorganisms in airborne microscopic dust or inhalation of oral secretions that contains high concentrations of microbes (Wu and Segal, 2018). Lots of studies on the pulmonary microbiota have suggested that multiple oral commensal bacteria can be found in the lower airways, which can influence the response of other commensal microbes in the host and environment (Pustelny et al., 2015; Dickson et al., 2017; Huffnagle et al., 2017). Three factors including microbial migration, microbial elimination, and the relative reproduction rate of its constituent members determined the composition of the lung microbiota (Invernizzi et al., 2020). The balance between migration and elimination might be disturbed during lung disease, leading to a shift in the lung microbiota, with those bacteria that have a competitive advantage gradually becoming dominant (Dickson and Huffnagle, 2015; O’Dwyer et al., 2016). However, it is not clear whether the pathogens that cause pneumonia are acquired by inhalation of oral pathogens or by the abnormal proliferation of pulmonary proteobacteria. In addition, a growing body of research suggests that the combination of the microbiota and their related metabolites might contribute to the understanding of mechanisms involved in the disease process (Qiao et al., 2019; Li et al., 2020). Disturbances in the composition and changes in the diversity of the pulmonary microbiota might be associated with dysregulated metabolite homeostasis (Li et al., 2020). However, there is no comprehensive study on the microbiota and metabolic characteristics of pneumonia induced by immunosuppression.

Cyclophosphamide is one of the most successful and widely utilized anticancer and immunosuppressive agent, which is used to prevent transplant rejection, treat some chronic autoimmune diseases and induce experimental immunosuppression (De Jonge et al., 2005; Emadi et al., 2009; Schulze et al., 2019; Berköz et al., 2021; Helsby et al., 2021). It is a phase-independent cytotoxic agent, which can inhibit humoral and cellular immunity (De Jonge et al., 2005; Emadi et al., 2009). Even today, 50 years after its synthesis, cyclophosphamide is still widely used as a chemo therapeutic agent. Among 1,000 selected compounds and antibiotics tested against 33 tumors, cyclophosphamide was the most effective molecule (Emadi et al., 2009). In previous scientific research, cyclophosphamide has also been frequently used in immunosuppressive animal models (Tian et al., 2016; Noh et al., 2019; Berköz et al., 2021). For these reasons, we chose cyclophosphamide as the most representative immunosuppressant to construct the immunosuppression model.

Our study suggested the source of pathogens of pneumonia in immunosuppressed rats and explored the relationship between pneumonia and metabolites in these immunosuppressed rats. The results of the present study could guide prevention and treatment strategies for pneumonia.

## Materials and Methods

### Animals

Male SD rats (weighing 180–200 g) were purchased from Vital River Laboratory Animal Technology Co., Ltd. (Beijing, China). These rats were raised under normal conditions (22 ± 2°C, 45 ± 5% humidity, and in a 12/12-h dark-light cycle). The animal experimental protocols were approved by the Animal Care and Use Committees of the Tianjin Institute of Environmental and Operational Medicine. The approval number is “IACUC of AMMS-04-2020-031.”

### Animal Study and Sample Collection

After 3 days of adaptive feeding, the rats were statistically randomized into four groups (*n* = 9 per group): control group (C), immunosuppression group (I), sterilized phosphate buffer saline (PBS) instillation control group (CP), and the PBS instillation immunosuppression group (IP). Rats in the I and IP groups received two intraperitoneal injections of cyclophosphamide (50 mg/kg) at 2-day intervals, and the C and CP groups were given matching normal saline (Huyan et al., 2011). One day after the last intraperitoneal injection, the rat’s tongue was pulled out using tweezers and 300 μL of sterilized PBS was instilled into the trachea through the oropharynx in both the CP and IP groups to simulate sucking oral secretions. All the rats were anesthetized with sodium pentobarbital prior to intratracheal injection (Tsubokura et al., 2016). The procedure was repeated on day 4 and day 7. One day after the third tracheal instillation, all the rats were euthanized under pentobarbital sodium.

For all the groups, the samples of blood were collected *via* cardiac puncture, 0.5 ml of blood was placed in sterile EDTA-anticoagulated tubes and counted in an automatic hematology analyzer (Shenzhen Mairui Corporation, China) (Huyan et al., 2011); their thymuses and spleens were removed, weighed, fixed in 4% paraformaldehyde, and stained with hematoxylin-eosin (H&E). The right upper lungs of all groups were also enucleated and stained with H&E. The rest of the lungs were stored directly at −80°C, and the right lower lungs of all groups were used for 16S rRNA gene sequencing. Then, the left lower lungs of the CP and IP groups were further used for metabolomics analysis. All the operations were carried out under sterile conditions.

### Sequencing and Analysis of 16S rRNA Gene of Pulmonary Microbiome

The cetyltrimethylammonium bromide (CTAB) method was applied to extract the total genomic DNA from lung samples and 1% agarose gels was used to analyze the concentrate and purity of the DNA. The target specific primers 515F (5′-GTGCCAGCMGCCGCGGTAA-3′) and 806R (5′-GGACTACHVGGGTWTCTAAT-3′) were used to amplify the V4 region. The 16S rRNA gene clone library was constructed by Novogene (Beijing, China) using the Illumina HiSeq 2500 platform.

According to QIIME (Version 1.7.0) (Kuczynski et al., 2011), the raw tags were processed under specific filtering conditions, and valid tags were obtained. The abundance information of operational taxonomic units (OTUs) was normalized using the sequence number standard which is corresponded to the sample with the smallest sequence number. Alpha diversities (Shannon, Simpson, Chao1, and ACE diversity indices) and beta diversities were evaluated to analyze the complexity and differences of the samples. Based on weighted UniFrac distance metric, principle coordinate analysis (PCoA) was performed to visualize separation of samples. The cladogram functionality of Linear discriminant analysis Effect Size (LEfSe) was applied to reveal the differences in microbiota composition between group I and C, and group IP and CP, with a linear discriminant analysis (LDA) score > 4.0. All of these data were calculated by QIIME (Version 1.7.0) and displayed by R software (Version 3.4.3) (Ma et al., 2018).

### Lung Metabolomics Analysis

According to a previously published protocol, organic metabolites were extracted sequentially (Li et al., 2020). The tissues (100 mg) were ground separately with precooled 70% methanol (500 μL), shaken for 6 min, and incubated on ice for 20 min. After centrifugation at 13,000 × *g* (4°C) for 12 min, the supernatants were transferred to new centrifuge tubes. The remaining precipitates were homogenized and centrifuged again, and then mixed with the previous supernatants. All the supernatants were rotated in the vacuum concentrator until dry, then reconstituted in 70% methanol and analyzed by liquid chromatography-tandem mass spectrometry (LC-MS/MS).

A Vanquish ultra-high-performance liquid chromatography (UHPLC) system and an Orbitrap Q Exactive HF-X mass spectrometer were used to perform the LC-MS/MS analyses at Novogene Genetics (Beijing, China). The raw data of UHPLC-MS/MS was extracted by the Compound Discoverer 3.1 (Thermo Fisher, Waltham, MA, United States) to obtain peak picking, peak alignment, and quantitation of each metabolite. The data files were matched with the mzCloud^1^ and database developed within mzVault (Version 2.3) to obtain accurate qualitative and relative quantitative results.

These metabolites were annotated with the Kyoto Encyclopedia of Genes and Genomes (KEGG) database^2^. Univariate analysis, which involved fold-change analysis, and multivariate analysis, which included partial least squares discriminant analysis (PLS-DA) and principal component analysis (PCA), were used to calculate the statistical significance of the difference in metabolites between the CP and IP groups. The relative content of the top 30 metabolites were shown using their *Z*-scores (standard score). Column charts, Volcano Plots, and *Z*-score Figures were made with the R software (Version 3.4.3) to visualize the differences of metabolites. The KEGG database was used to perform the pathway enrichment analysis of those various metabolites.

### Statistical Analysis

The statistical analysis of white blood cells was performed using GraphPad Prism version 7.0 (GraphPad Software Inc., La Jolla, CA, United States). Student’s test, Wilcoxon’s test or Analysis of variance (ANOVA) were used to perform the statistical analysis of the quantitative multiple group comparisons. The relationship between the lung microbiome and the potential influencing factors in matching metabolic profiles were explored using Redundancy analysis (RDA) with Canoco 5 software (Forsberg et al., 2014) and pairwise Pearson’s correlations with the Hmisc package in R software (Version 3.4.3) (Ma et al., 2018). Networks using pairwise Pearson’s correlations were constructed on the platform of Gephi v0.9.2 (Bastian et al., 2009). When *P*-value was less than 0.05, the results were considered statistically significant.

## Results

### Evaluation of the Immunosuppression Model

**FIGURE 1:**
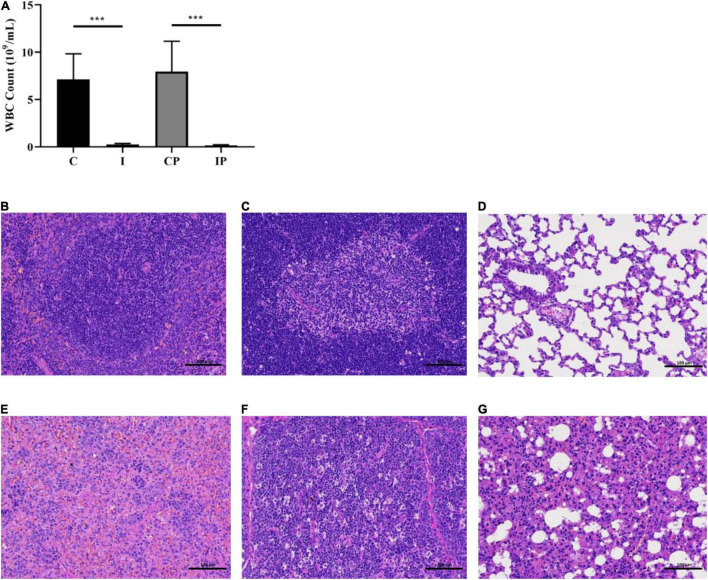
Blood WBC cell count of rats and H&E staining of spleen tissue, thymus gland tissue, and lung tissue. WBC cell count **(A)**. H&E staining of spleen tissue **(B)**, thymus gland tissue **(C)**, and lung tissue **(D)** of rats in the CP group. H&E staining of spleen tissue **(E)**, thymus gland tissue **(F)**, and lung tissue **(G)** of rats in the IP group. Scale bar in each panel, 100 μm. ***p-value < 0.001 versus the control group, *n* = 9 in (C, I, CP, and IP). WBC, white blood cell; H&E, hematoxylin and eosin; CP, sterilized phosphate buffer saline (PBS) instillation control group (CP); IP, PBS instillation immunosuppressed group.

White blood cells were detected and the spleen tissue and thymus tissue histology were evaluated with H&E staining to visualize the difference in the number of immune cells. The number of white blood cells in the I group was obviously lower than that in the C group, and the same results could be seen between group IP and group CP (Figure 1A). The differences in the relative weights of spleen and thymus were significant in group C compared with group I and group CP compared with group IP (*P* < 0.05) (Supplementary Figure 1) (Kluxen, 2019). It can be seen that the splenic tissue in groups C and CP had normal red and white marrow structure, intact organization and tight arrangement (Supplementary Figures 2A,C), and lymphocytes were mainly present in splenic vesicles (Figure 1B and Supplementary Figure 3A). In contrast, in groups I and IP, the white marrow was relatively small in size, irregular in morphology, and the marginal zone disappeared (Supplementary Figures 2E,G), and some of the lymphocytes disappeared from nuclear consolidation and were phagocytosed by histiocytes (Figure 1E and Supplementary Figure 3D). In groups I and IP, the thymic lobules were incompletely divided, with indistinct cortical and medullary boundaries and markedly diminished basophilia (Supplementary Figures 2F,H), and a large number of apoptotic lymphocytes at the lobular margins, with loss of nuclear consolidation and phagocytosis by histiocytes (Figure 1F and Supplementary Figure 3E). By contrast, these changes were not observed in the C or CP group (Figure 1C and Supplementary Figures 2B,D, 3B). Our experimental results are consistent with those of previous immunosuppression models (Noh et al., 2019; Berköz et al., 2021).

H&E staining was also used to evaluate the histopathological changes in lung tissue following immunosuppression. The lung tissue of the C group and the I group (Supplementary Figures 3C,F) and the CP group (Figure 1D) maintained an intact terminal bronchial and alveolar epithelium without inflammation. While in the IP group, the alveolar wall thickened slightly to moderately, the alveolar cavity was narrow, infiltration of pulmonary macrophages can be seen, and there were some fibrous substances in the airway cavity (Figure 1G) (Li et al., 2020). The above results indicated that we had established an immunosuppressed rat model, and there was inflammatory injury in the lungs of the IP group.

### Effect of Immunosuppression on the Community Richness and Diversity of the Pulmonary Microbiome

**FIGURE 2:**
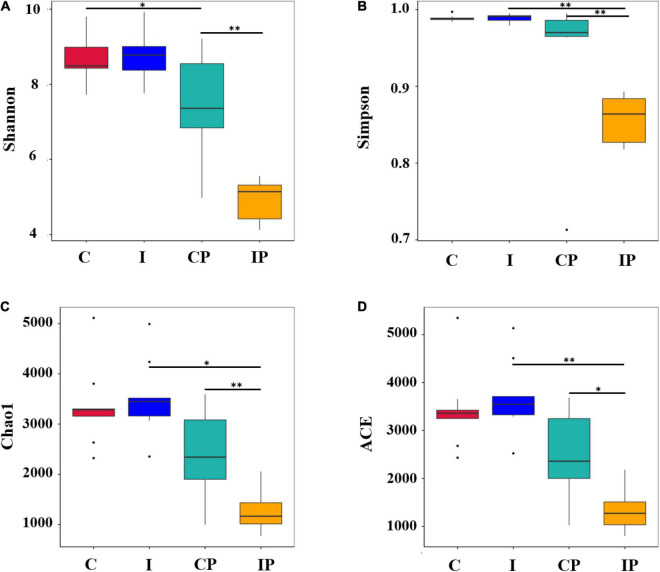
Alpha diversity of microflora. The diversity **(A,B)** and richness **(C,D)** of bacteria are displayed by boxplots for the C (red), I (blue), CP (light blue), and IP (yellow) groups. The median, largest and smallest observations are shown in the figure. The statistical significance of the alpha diversity analysis was determined by using a Wilcoxon rank sum test. The *P*-values of ^∗^ and ^∗∗^ are <0.05 and 0.01, respectively, *n* = 9 in (C, I, CP, and IP). C, control group; I, immunosuppressed group; CP, sterilized phosphate buffer saline (PBS) instillation control group; IP, PBS instillation immunosuppressed group.

The alpha diversity metric was applied to calculate the community diversity and richness in the pulmonary microbiome. The scores of the diversity estimator Shannon and Simpson in the IP group were much lower than that in the CP group (Figures 2A,B). However, no significant difference was observed between group C and group I. Meanwhile, the Chao1 index and ACE index showed that the lung microbial community richness in the IP group was significantly lower than that in the CP group (Figures 2C,D). However, there was no obvious difference between the C group and I group. In addition, the Simpson index, Chao1 index, and ACE index in the IP group were much lower than those in the I group. By contrast, no differences of these indexes were observed between group C and group CP. These results indicated that the diversity and richness of the pulmonary microflora in the IP group were more likely to change.

### Effects of Immunosuppression on Lung Microbiome Composition

In our study, 12,740 OTUs were found in the open sequence of the SILVA 138 repository. Of these, 12,272 (96.33%) OTUs were noted in the database. At the kingdom, phylum, class, order, family, genus, and species level, the proportions of annotated OTUs were 96.33, 82.32, 79.90, 72.83, 60.83, 38.54, and 7.20%, respectively.

The most abundant bacteria in the group were investigated at the phylum, family, and genus levels. At the phylum level, the dominant phylum in the rat pulmonary microbiota were *Firmicutes*, *Proteobacteria*, *Actinobacteriota*, and *Bacteroidota*, with a total abundance close to 80%. There was no significant difference in the relative levels of *Firmicutes*, *Actinobacteriota*, and *Bacteroidota* between group C and group I; however, the level of *Proteobacteri*a in group I was higher than that in group C (*P* < 0.05). Compared with group CP, the relative abundance of *Firmicutes* and *Proteobacteria* in group IP was higher (*P* < 0.001), while the relative abundance of *Actinobacteria* (*P* < 0.05) and *Bacteroidota* was lower (*P* < 0.001).

**FIGURE 3:**
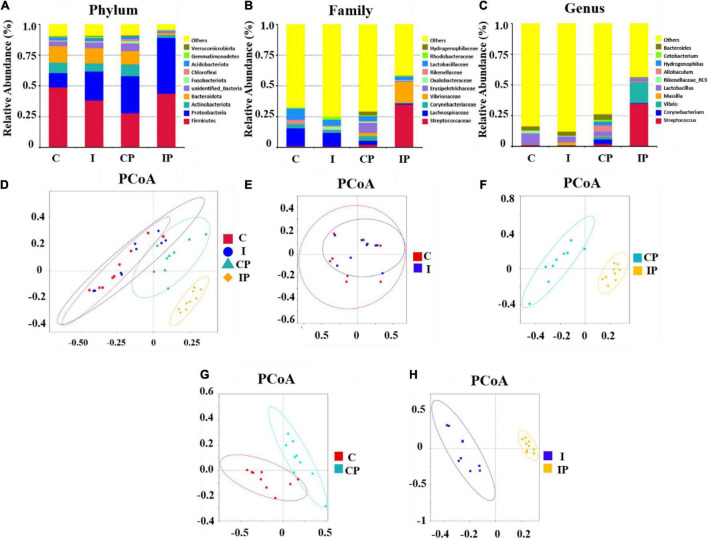
Composition of the pulmonary microbiota was modified after intratraceal PBS administration in immunosuppressed rats. Composition of bacteria in different groups at the phylum level **(A)**, family level **(B)**, and genus level **(C)**, respectively. The PCoA plot explained the maximum variance between all samples **(D)**, groups C and I **(E)**, CP and IP **(F)**, C and CP **(G)**, and I and IP **(H)**, respectively. *N* = 9 in (C, I, CP, and IP). PCoA, principal coordinates analysis; OUT, operational taxonomic unit; C, control group; I, immunosuppressed group; CP, sterilized phosphate buffer saline (PBS) instillation control group; IP, PBS instillation immunosuppressed group.

In the CP group, in comparison with the C group, the relative abundance of *Proteobacteria* increased, but *Firmicutes* decreased. Compared with group I, group IP showed an increase in the relative abundance of *Proteobacteria* and a decrease of *Bacteroidota* (Figure 3A). The distribution of the main microbiota at the family and genus level was further analyzed. At the family level (Figure 3B), the relative abundance of *Streptococcaceae* in the IP group (34.91%) was much higher than that in the C group (1.03%), I group (0.76%), and CP group (2.14%) (*p* < 0.05). At the genus level (Figure 3C), the relative amount of *Streptococcus* was significantly higher in the IP group (34.88%) than in the C group (1.00%), I group (0.71%), and CP group (2.10%) (*p* < 0.05).

A PCoA plot was used to show the structure of the lung microbiome (Figure 3D). There was no significant difference in the structure of the C group and I group (Figure 3E). By contrast, the microbiome of the CP and IP groups were successfully separated, with a contribution of 46.71 and 21.31% for PC1 and PC2 principal components, respectively (Figure 3F). Additionally, compared with the C group, the CP group showed 43.49% variance (Figure 3G). However, compared with the I group, the IP group showed 53.73% variance (Figure 3H).

**FIGURE 4:**
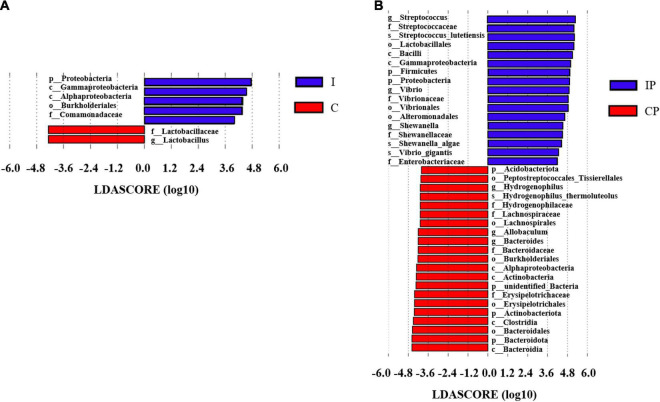
The differentially enriched microbiota in rats exposed to cyclophosphamide and oropharyngeal bacteria. Varying degrees of bacterial abundance (LDA > 4) shown by LEfSe analysis in group C compared with group I **(A)** and group CP compared with group IP **(B)**. *N* = 9 in (CP and IP). LEfSe, linear discriminant analysis effect size; LDA, linear discriminant analysis; C, control group; I, immunosuppressed group; CP, sterilized phosphate buffer saline (PBS) instillation control group; IP, PBS instillation immunosuppressed group.

The LEfSe analysis was performed on the relative abundance of bacteria between the different groups, and values with LDA scores greater than 4 or less than −4 were selected to show the most obviously enriched bacteria in each group. The relative abundance of *Proteobacteria* at the phylum level and the relative abundance of *Comamonadaceae* at the family level showed an increase in group I in comparison with group C (Figure 4A). Notably, the relative abundances of *Proteobacteria* and *Firmicute* at the phylum level; the relative abundance of *Streptococcaceae*, *Vibrionaceae*, *Enterobacteriaceae*, and *Shewanellaceae* at the family level; and the relative abundance of *Streptococcus*, *Shewanella*, and *Vibrio* at the genus level, were increased in the IP group compared with that in the than CP group (Figure 4B). Differences in the relative abundance of the above-mentioned bacteria between groups could also be obtained by one-way ANOVA multiple comparisons (Supplementary Figure 4).

### Effect of Immunosuppression on the Lung Metabolome

**FIGURE 5:**
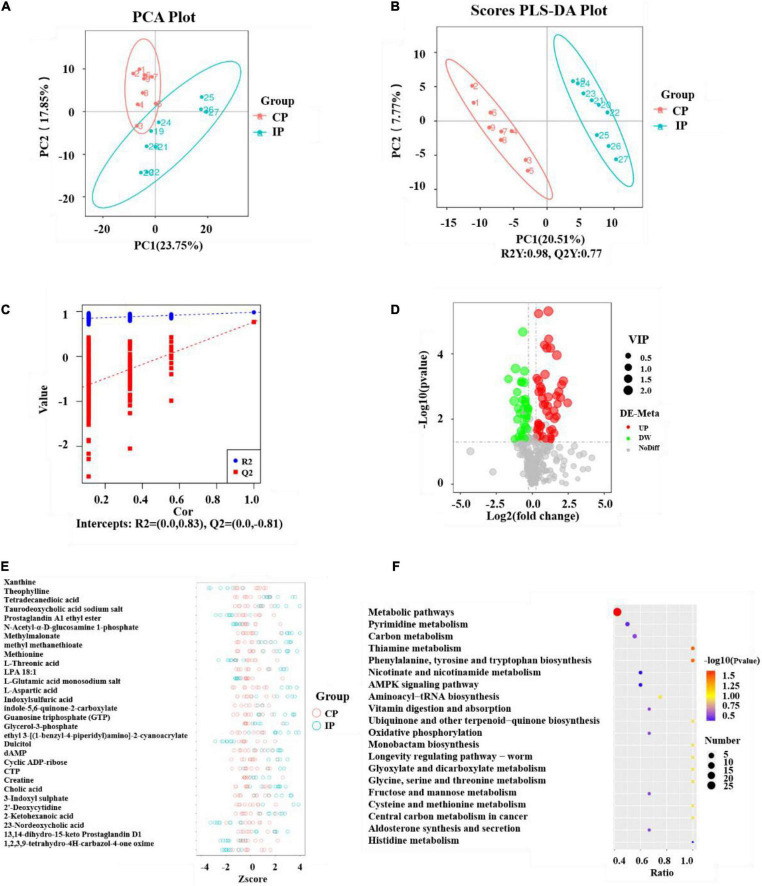
Analyses of the metabolites and pathway after intratraceal PBS administration between the CP and IP groups. PCA scatter plot of metabolite distribution **(A)**. PLS-DA analysis of the metabolite profile **(B)** and PLS-DA model test plots **(C)**. They showed the differentiation between the IP and CP groups, with R2Y = 0.98, Q2Y = 0.77, R2 = (0.0, 0.83), and Q2 = (0.0, –0.81). Volcano Plot depicting the differential metabolites between the CP and IP groups **(D)**. Metabolites meeting VIP > 1.0, FC > 1.2 or FC < 0.833, with a *P*-value < 0.05 were considered differential metabolites. The *Z*-score figure of the top 30 relative content of metabolites on the same level **(E)** and pathway analysis **(F)** of the lung tissue between the IP and CP groups. *N* = 9 in (CP and IP). VIP, variable importance projection; FC, fold-change; PCA, principal components analysis; PLS-DA, partial least squares discriminant analysis; CP, sterilized phosphate buffer saline (PBS) instillation control group; IP, PBS instillation immunosuppressed group.

The metabolite spectrum of lung tissues in the CP and IP groups were further analyzed using LC-MS. As shown in Figure 5A, metabolites were well separated in the CP and IP groups, with the principal components PC1 and PC2 explaining 23.75 and 17.85% of the variation, respectively. Next, the metabolite responsible for the differences between the different experimental groups were further analyzed using the PLS-DA model. The PLS-DA scores indicated that the CP and IP groups were dispersed into two separated regions (Figure 5B). The predictive ability values and goodness of fit [IP vs. CP group: R2Y = 0.98, Q2Y = 0.77, R2 = (0.0, 0.83), Q2 = (0.0, −0.81)] showed that the PLS-DA model had a good predictive ability and a satisfactory fit (Figures 5B,C).

In this study, variable importance projection (VIP) was used as a threshold to further screen for metabolites whose levels differed between the CP and IP groups. Metabolites meeting VIP > 1.0, fold-change (FC) > 1.2 or FC < 0.833, with a *P*-value < 0.05 were considered differential metabolites (Haspel et al., 2014; Heischmann et al., 2016) (Figure 5D). A total of 86 differential metabolites were identified between group IP and group CP. Compared with their levels in the CP group, 51 metabolites were elevated and 35 metabolites were decreased in the IP group. To further observe the differences in metabolites, qualitative and quantitative analyses of the major metabolites identified in each group were performed. The *Z*-score, which is used to measure the relative content of metabolites on the same level, identified the top 30 metabolites (Figure 5E).

The KEGG database was used to analyze the different metabolites and metabolic pathways were constructed and analyzed to better identify related metabolic pathways involved in immunosuppressive effects. The analysis indicated that these metabolites are involved in Metabolic pathways; phenylalanine, tyrosine, and tryptophan biosynthesis; and Thiamine metabolism (Figure 5F).

### Potential Relevance of Lung Microbiota to Metabolites

**FIGURE 6:**
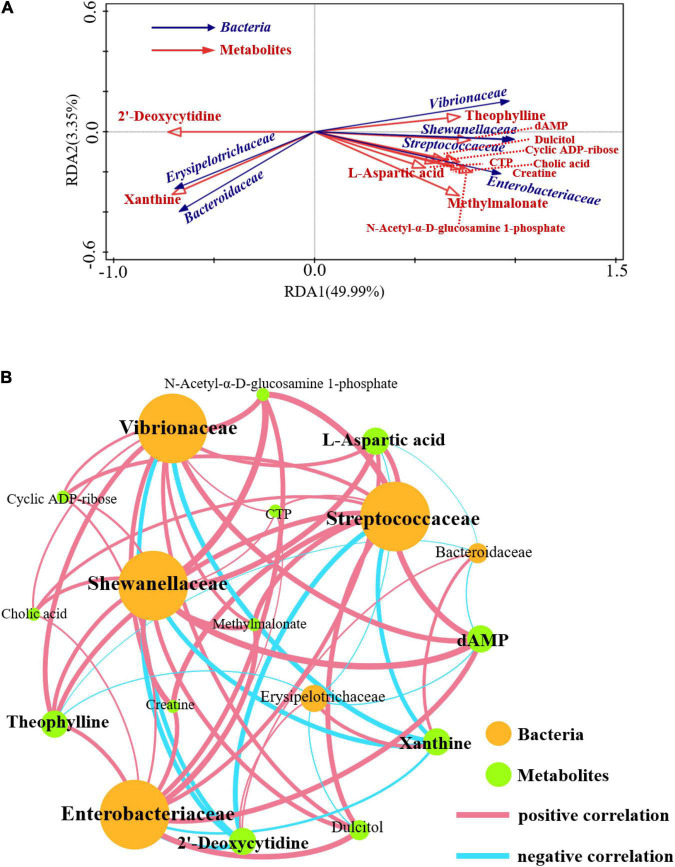
Correlations between the differential metabolites and microbiome. Redundancy analysis (RDA) between the bacterial community and lung microbiomes **(A)**. Effective variables were selected based on variance inflation factors (VIF). Correlations between the composition of the bacterial community at the family level with the lung microbiome **(B)**. Nodes are colored according to metabolites or bacteria. Edges represent significant correlations between two nodes (*P* < 0.05). The node size is weighted according to the number of connections and the thickness of the edges is weighted according to the Pearson correlation coefficient. *N* = 9 in (CP and IP).

The functional correlation of changes in lung microorganisms and metabolites between the CP and IP groups was explored using redundancy analysis (RDA) and calculation of Pearson correlation coefficients (*p* < 0.05) (Figures 6A,B). The results showed that the relative amount of some bacterial families correlated significantly with the content of various metabolites in the host. For example, levels of L-aspartate, creatine, methyl malonate, DAMP, and *N*-acetyl-α-D-glucosamine 1-phosphate correlated positively with the relative abundance of several families, including *Streptococcaceae*, *Enterobacteriaceae*, and *Shewanellaceae*. Conversely, L-aspartic acid and dAMP levels correlated negatively with the relative abundance of *Erysipelotrichaceae*. Overall, our results suggest that changes in pulmonary microbiota correlate with changes in metabolites.

## Discussion

In this study, we initially constructed an immunosuppression model *via* intraperitoneal injection of cyclophosphamide in rats. We then instilled sterilized PBS into the trachea through the oropharynx to replace oropharyngeal bacteria into the lower respiratory tract, which was similar to that observed in immunosuppressed humans who accidentally sucked in oral secretions. We detected the immune status of the rats according to the immune-related white blood cells and the histology of the thymus and spleen. The results presented that the leukocyte count of rats in the immunosuppressed group was obviously lower than that in the control group, which was in line with the study of Huyan et al. (2011). Compared with the H&E staining results of the spleen and thymus tissues in the healthy rats, the number of immune cells in the immunosuppressed group decreased obviously. This observation was consistent with that of previous studies (Lee et al., 2018; Noh et al., 2019), indicating that the immunosuppression model was successfully constructed. The lung histopathology according to the H&E staining results indicated that there was inflammatory injury in the lungs in the IP group, which was not obvious in the other groups. This observation is similar to previous studies, which showed that immunosuppressed patients are more likely to develop pneumonia than healthy people (Di Pasquale et al., 2019).

To our best knowledge, this was the first time that 16S rRNA sequencing and LC-MS metabolomics techniques have been used to study the effect of immunosuppression on lung microbiota and its metabolism. Our findings revealed that the diversity and richness of the pulmonary microflora in the immunosuppressed group were more susceptible to exogenous bacteria. There was no obvious change in the distribution of microflora between the C group and I group. However, after the introduction of oropharyngeal bacteria by PBS, the difference in the bacterial distribution between the IP and CP groups was significant (*P* < 0.05). Although there were differences between group C and CP, it was clear that the difference between group I and IP was more obvious. These results suggested that the flora of pneumonia in immunosuppressed patients was caused by inhalation of oropharyngeal pathogens rather than the abnormal proliferation of proteobacteria in the lungs. Interestingly, we also observed that, at the family level, the relative abundance of *Streptococcaceae* and *Enterobacteriaceae* were increased to a greater extent in the IP group than in the CP group. This suggested that immunosuppression might increase susceptibility to *Streptococcaceae* and *Enterobacteriaceae* infection and cause pneumonia. This result is in agreement with previous reports, in which *Streptococcus pneumoniae* was the most frequently detected pathogen, followed by *Enterobacteriaceae* in both immunocompromised and immunocompetent groups (Polverino et al., 2010; Putot et al., 2016). In addition, the air in which we live contains bacteria, which can affect the composition of bacteria in the oropharynx. If further inhaled by immunosuppressed patients, they are likely to cause pneumonia. This might be the reason why immunosuppressed patients are more likely to suffer from pneumonia.

The migration of microbiota from the upper respiratory tract to the lower respiratory tract is mainly promoted by subclinical inhalation, which occurs in patients with depressed sensorium and also in normal adults during deep sleep (Huxley et al., 1978; Gleeson et al., 1997). When local defense is weakened or overwhelmed, the aspirated bacteria are not effectively cleared and obvious clinical infections occur (Huxley et al., 1978; O’Dwyer et al., 2016). Host inflammatory cells are responsible for eradication of pathogens and the type and number of effector cells are associated with certain features of the microbiome. Segal et al. (2013) demonstrated increased community abundance of oral bacteria associated with higher levels of lymphocyte and neutrophil inflammation. We speculate that the immunosuppressive effect of cyclophosphamide on immune cells such as neutrophils and lymphocytes weakens their ability to remove foreign bacteria entering the lungs, thus making it easier for mistakenly swallowed bacteria to survive and lead to changes in the flora of the patients’ lungs (O’Dwyer et al., 2016). At the same time, whether immunosuppression interferes with oral microorganisms needs to be carefully considered. In recent years, several studies have highlighted the role of the microbiome in the pathogenesis of autoimmune diseases (Belkaid and Hand, 2014; Nikitakis et al., 2017; De Luca and Shoenfeld, 2019). At the same time, other autoimmune diseases (i.e., systemic sclerosis, Sjögren’s syndrome and anti-phospholipid syndrome) also share modifications of the microbiome in the intestinal tract and oral flora (Belkaid and Hand, 2014; Nikitakis et al., 2017; De Luca and Shoenfeld, 2019). As far as we know, there are few studies on the changes of oral microorganisms under the condition of immunosuppression in experimental animals. A larger project is still needed to better define the distribution of oral microbiota and their role in pulmonary bacterial infection under immunosuppressive conditions.

Bacterial fitness is closely related to the ability of bacteria to absorb nutrients or amino acids provided by their respective host ecological niches, because they require energy and carbon to grow and replicate. Consequently, pathogenic bacterial physiology must be adapted to these different physiological conditions to ensure the expression of adaptive and virulence factors (Hartel et al., 2012). The microbial community changes dynamically depending on the lung condition, followed by pneumococcal infection (Chen et al., 2020). In this paper, we found that L-Aspartic acid, Methylmalonate, *N*-Acetyl-α-D-glucosamine 1-phosphate, Theophylline, and dAMP, which are the main intermediates of metabolic pathways, were positively related to the abundance of *Streptococcaceae* and *Enterobacteriaceae*. They are likely to affect the survival of bacteria through changes in metabolic pathways. *Streptococcus pneumoniae* has a nutritional deficiency for arginine, which is metabolized by the arginine deiminase system (ADS), producing 1 mole of Ornithine and CO_2_, 2 moles of Ammonia and 1 mole of ATP per mole of Arginine (Abdelal, 1979). Arginine is the substrate for the arginine-ornithine antiporter, which is required for the maintenance of pneumococcal fitness (Schulz et al., 2014). L-aspartic acid is beneficial to the biosynthesis of Arginine, thus it might be beneficial to the survival of *S. pneumoniae*. Moreover, Aspartic acid was also identified as the growth promoter of *Enterobacteriaceae* (Ohsugi et al., 1993), thus its increase might also be a favorable factor for *Enterobacteriaceae*. In addition, differential metabolome and metabolic pathway analysis showed that L-serine, L-leucine, and L-valine protected rats from *Klebsiella pneumoniae* lung infection, enhanced macrophage phagocytosis, and were key metabolites that are positively associated with survival rats with a lung infection. Methylmalonate can lead to the degradation of Valine and Leucine, and both Creatine and L-Aspartic acid can lead to the metabolism of Serine. These may be beneficial for the survival of the *K. pneumoniae* (*Enterobacteriaceae*). By contrast, the interaction of the pulmonary microbiota with the airway immune system suggests that disruption of the pulmonary microbiota might be a pathogenic pathway leading to lung injury (Lynch, 2016; Wang et al., 2017). 2′-deoxycytidine, which is the main intermediate of Pyrimidine metabolism, and Xanthine, which is the main intermediate of Purine metabolism, were both negatively associated with the abundance of *Streptococcaceae* and *Enterobacteriaceae*. It is reported that purines and pyrimidines could regulate signals through purinergic receptors, which are associated with lung injury (Lazarowski and Boucher, 2009). According to the report of Li et al. (2014) purine metabolic disorder is associated with decreased lung function. Decreases of 2′-Deoxycytidine and Xanthine might lead to the metabolic disorder of purine and pyrimidine, which can lead to lung injury. Nevertheless, the exact interactions between lung microbiota and metabolite changes require further investigation.

In summary, this study investigated the effects of immunosuppression on the composition of the rat lung microbiota and lung metabolites. An immunosuppression model was established with cyclophosphamide, oropharyngeal saliva inhalation was simulated with sterilized PBS, flora was analyzed by 16S rRNA gene sequencing, and metabolites were analyzed by LC-MS metabolites. The results showed that sterilized PBS had a significant effect on the composition of the lung microbiota in the immunosuppressed group of rat compared with the control group. The percentage of *Streptococcus* and *Enterobacteriaceae* bacteria in lung tissue was higher in the immunosuppressed group after inhalation of sterilized PBS compared with the immunosuppressed group. Lung L-aspartic acid was elevated and 2′-Deoxycytidine and Xanthine were decreased in the immunosuppressed group after aspiration with sterile PBS compared with the control group. Correlation analysis showed a significant correlation between the changed flora and the changed metabolites. On the one hand, the increase of L-Aspartic acid may favor the survival of *Streptococcus* and *Enterobacteriaceae* bacteria. On the other hand, the decrease of 2′-Deoxycytidine and Xanthine may lead to disturbance of purine and pyrimidine metabolism, thus causing lung damage. Based on the experimental results, we hypothesize that the pathogen of immunosuppression-associated pneumonia may arise from inhalation of oropharyngeal pathogens rather than abnormal proliferation of proto-bacteria in the lung, and that there is a correlation between the proliferation of pathogens and changes in the associated metabolites. In summary, this study provides a new perspective on the disruption of the pulmonary flora and its associated metabolites, one of the pathogenic mechanisms of immunosuppressive pneumonia. It might have important reference value for clinical prevention and treatment of immunosuppression-associated pneumonia.

## Data Availability Statement

The datasets presented in this study can be found in online repositories. The accession number can be found below: https://www.ncbi.nlm.nih.gov/, PRJNA743947.

## Ethics Statement

The animal study was reviewed and approved by the Animal Care and Use Committees of the Tianjin Institute of Environmental and Operational Medicine. Written informed consent was obtained from the owners for the participation of their animals in this study.

## Author Contributions

JL and DY: conceptualization. HD, RT, LW, and YS: investigation. ZC: formal analysis. HD and DY: writing – original draft. MJ, JY, and HL: writing – review and editing. All authors contributed to the article and approved the submitted version.

## Conflict of Interest

The authors declare that the research was conducted in the absence of any commercial or financial relationships that could be construed as a potential conflict of interest.

## Publisher’s Note

All claims expressed in this article are solely those of the authors and do not necessarily represent those of their affiliated organizations, or those of the publisher, the editors and the reviewers. Any product that may be evaluated in this article, or claim that may be made by its manufacturer, is not guaranteed or endorsed by the publisher.

## Footnotes

1
https://www.mzcloud.org/

2
https://www.genome.jp/kegg/pathway.html
