# Supplementary material for: The Effects of Immunosuppression on the Lung Microbiome and Metabolites in Rats
Source: Front Microbiol. 2022 Feb 14;13:817159. doi: 10.3389/fmicb.2022.817159 (PMC8882871; doi:10.3389/fmicb.2022.817159)
Supplement: Supplementary file 1 [file Data_Sheet_1.pdf]

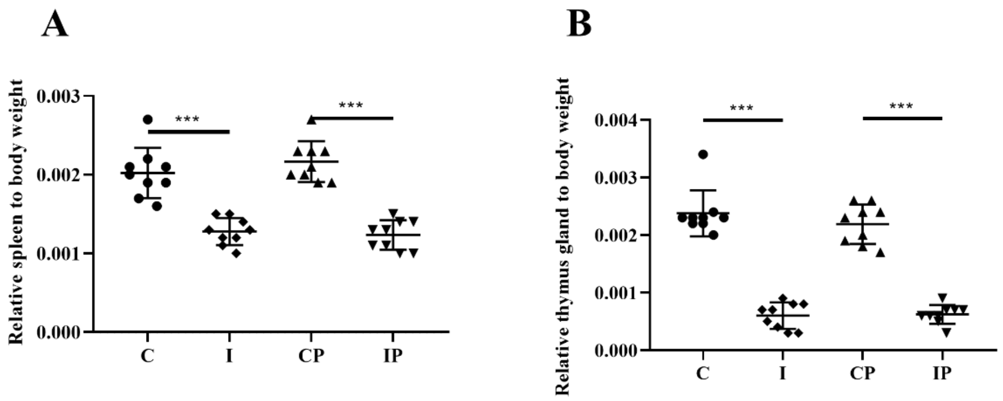

Supplementary Fig. 1. Scatter plotting of relative weight of spleen and thymus. Relative weight of spleen (A); Relative weight of spleen thymus gland (B). Relative weight is the ratio of the weight of an organ to its body weight in an experimental animal. *** p-value < 0.001, n = 9 in (C, I, CP, IP); C, normal control group; I, immunosuppressed group; CP, sterilized phosphate buffer saline (PBS) instillation control group (CP); IP, PBS instillation immunosuppressed group.

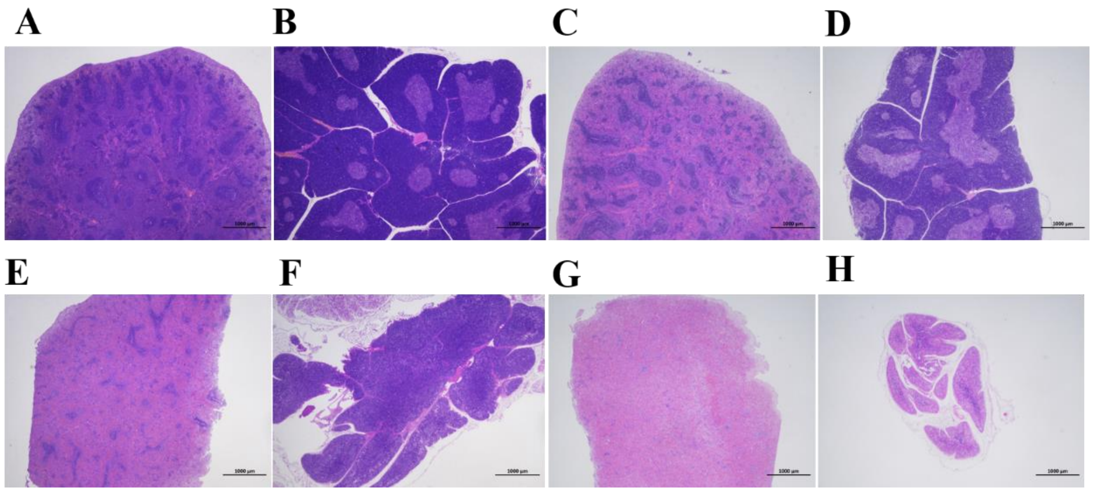

Supplementary Fig. 2. H&E staining of spleen tissue and thymus gland tissue. H&E staining of spleen tissue (A), thymus gland tissue (B) of rats in the C group. H&E staining of spleen tissue (C), thymus gland tissue (D) of rats in the CP group. H&E staining of spleen tissue (E), thymus gland tissue (F) of rats in the I group. H&E staining of spleen tissue (G), thymus gland tissue (H) of rats in the IP group. Scale bar in each panel, 1000μm. N = 9 in (C, I, CP, IP). H&E, hematoxylin and eosin; C, normal control group; I, immunosuppressed group; CP, sterilized phosphate buffer saline (PBS) instillation control group (CP); IP, PBS instillation immunosuppressed group.

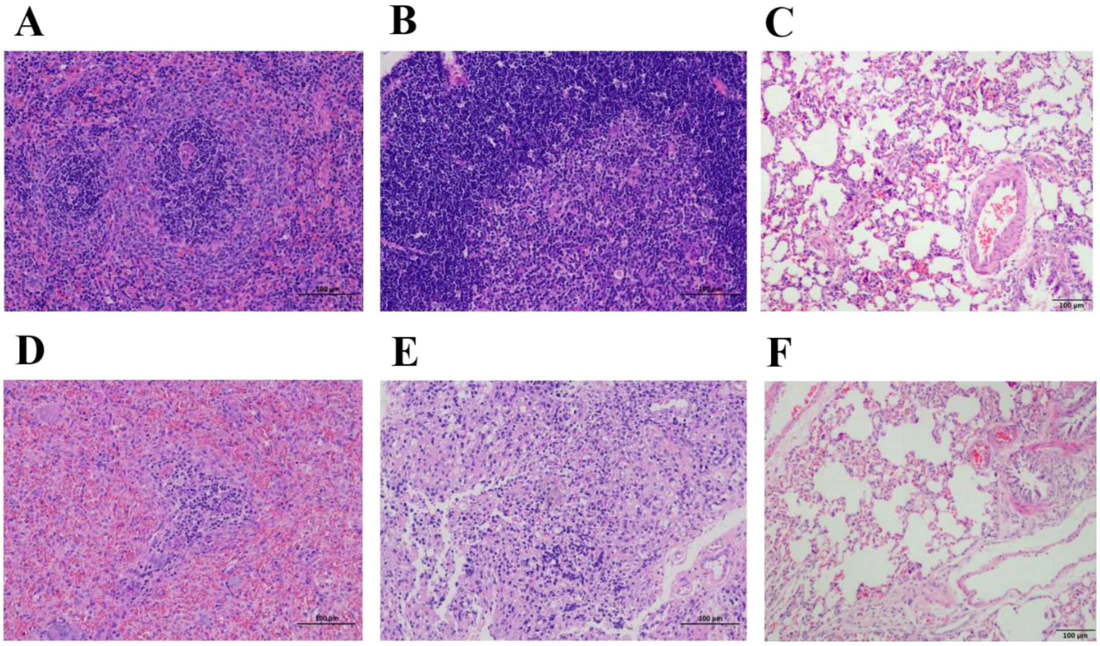

Supplementary Fig. 3. H&E staining of spleen tissue, thymus gland tissue, and lung tissue of group C and I. H&E staining of spleen tissue (A), thymus gland tissue (B), and lung tissue (C) of rats in the C group. H&E staining of spleen tissue (D), thymus gland tissue (E), and lung tissue (F) of rats in the I group. N = 9 in (C, I). Scale bar in each panel, 100 μm. H&E, hematoxylin and eosin; C, normal control group; I, immunosuppressed group.

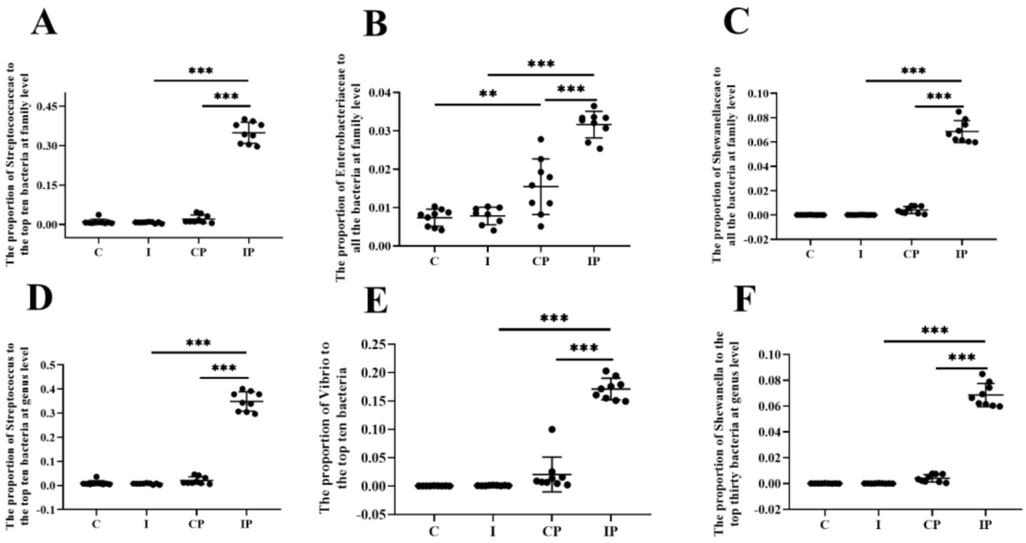

Supplementary Fig. 4. The scatter plot of the proportion of bacteria in each group. At the family level, the proportion of *Streptococcaceae* in the top 10 bacteria in each group(A), the proportion of *Enterobacteriaceae* in each group(B), the proportion of *Shewanellaceae* in each group (C). At the genus level, the proportion of *Streptococcus* (D), *Vibrio* (E) in the top ten bacteria in each group, and the proportion of *Shewanella* in the top 30 bacteria in each group(F). ***p-value < 0.01, *** p-value < 0.001, n = 9 in (C, I, CP, IP); C, normal control group; I, immunosuppressed group; CP, sterilized

phosphate buffer saline (PBS) instillation control group (CP); IP, PBS instillation immunosuppressed group.
